# Allelopathic and Autotoxic Effects of *Medicago sativa*—Derived Allelochemicals

**DOI:** 10.3390/plants8070233

**Published:** 2019-07-18

**Authors:** Bimal Kumar Ghimire, Balkrishna Ghimire, Chang Yeon Yu, Ill-Min Chung

**Affiliations:** 1 Department of Applied Life Science, Konkuk University, Seoul 05025, Korea; 2 Division of Plant Resources, Korea National Arboretum, Pocheon 11186, Korea; 3 Department of Agriculture Life Sciences, Kangwon National University, Chuncheon 24341, Korea

**Keywords:** allelopathic properties, callus growth, alfalfa leaf extracts, saponins, phenolic compounds, nuclear magnetic resonance

## Abstract

In this study, the allelopathic properties of *Medicago sativa* on different weeds were investigated under in vitro conditions. The compounds involved in the autotoxicity of *M. sativa* were analyzed using high-performance liquid chromatography. The extracts of all concentrations inhibited the growth of the calluses of *Digitaria ciliaris, Chenopodium album*, *Amaranthus* *lividus, Portulaca oleracea*, and *Commelina communis*. Six allelopathic compounds in alfalfa were identified and quantified, and the most predominant phenolic compounds were salicylic acid and *p*-hydroxybenzoic acid. Various concentrations (10^−2^, 10^−3^, and 10^−5^ M) of all the tested phenolic compounds exerted inhibitory effects on callus fresh weight. Rutin, salicylic acid, scopoletin, and quercetin significantly inhibited alfalfa seed germination. Of the seven identified saponins, medicagenic acid saponins exhibited the highest autotoxic effect and significantly lowered seed germination rate. Principal component analysis showed that the phenolic compounds and saponin composition significantly contributed to the different variables. The highly phytotoxic properties of the alfalfa-derived phenolic compounds and saponins indicate that these phytochemicals can be a potential source of bioherbicides.

## 1. Introduction

Numerous methods for weed management in different systems have been developed over the decades. Of these, herbicide use and hand and mechanical weeding are the most reliable weed control methods [1,2,3]. Herbicides use is increasingly being adopted for weed control and for increasing crop production [4]. However, herbicide-resistant weeds are the major constraint to weed control in modern agriculture. Additionally, hand weeding and mechanical weed control methods are expensive and labor intensive [5,6,7]. Furthermore, weed control via chemical herbicide use reportedly negatively impacts the environment and human health [8,9,10,11,12,13,14]. Extensive exposure to chemical herbicides may cause numerous health issues such as reproductive problems, neurologic impairment, pancreatic cancer, immune malfunction, and silicosis [15]. Furthermore, herbicides are unaffordable to small-scale farmers and unsuitable for organic farming. These challenges associated with the use of herbicides and mechanical weed control methods in land cultivation make it imperative to develop novel, environmentally friendly methods. The global interest in alternative, sustainable, and innovative technologies for weed management that not only reduce chemical herbicide use but are also environmentally and health-friendly and decrease agricultural production cost is increasing.

Allelopathy is defined as the direct/indirect harmful/beneficial effect of one plant on another via the production of chemical compounds that escape into the environment. Allelochemicals are generally secondary plant metabolites, which are part of a wide range of chemicals synthesized by plants, not directly involved in plant growth and development. Nowadays, allelopathic plants are being used for weed control and natural herbicide development. The environmental- and biodiversity-conserving nature of allelopathic weed control has made it more popular than mechanical methods and chemical herbicides. Some plant species exude useful biochemicals (also called allelochemicals or phytochemicals) that inhibit/suppress seed germination and weed growth [16,17]. These phytochemicals are generally phenolics (such as tannins), alkaloids, steroids, terpenes, saponins, and quinones that affect the growth and development of certain plant species [18]. The mechanism of action of some allelochemicals is similar to that of chemical herbicides, making them ideal for weed management. The allelochemicals produced by donor plants are often detrimental to their own species—an autotoxic effect [19]. These phytochemicals are totally/partially water soluble, making them more environmentally friendly than chemical herbicides [18,20]. They are characterized by higher O_2_- and N_2_-rich molecules and few halogen substitutes [19]. Allelopathy has a positive effect on soil, as it improves nutrient availability to crops by increasing microbial activity [21,22]. Allelochemicals are exuded from different plant parts at different ratios, and their exudation varies within different cultivars [23,24].

Both the allelopathic and autotoxic nature of plant species are useful for the invasion, distribution, and abundance of plants within the plant community [25,26,27,28,29,30]. The degree of allelopathy depends on disease, nutrition, insects, competition [31,32], biotic factors such as nutrients level, and abiotic factors such as temperature, irradiation, draught, and pH [33,34]. Allelopathic and autotoxic compounds are released from donor plants as volatiles, roots exudates, or foliage leachates, and contain several secondary metabolites such as flavonoid phenolics [35], ketones, aldehydes, terpenoids, lactones, cinnamic acid, and quinines [36]. If these compounds are released into the soil by any plant, they may exert toxic effects on neighboring plants or aid the production of new toxins by soil microbes [37]. The mechanism of action of allelochemicals in neighboring plants depends on their effects, which could be synergistic, additive, or antagonistic [38,39,40].

*Medicago sativa* L. (Leguminosae), commonly called alfalfa, is a perennial herb mostly grown in America, Europe, and Asia [41]. It is used as a source of protein [42,43], fat, and crude fiber [44] and as hay, silage, and pasture [45,46]. Traditionally, its dried leaf is used to lower cholesterol levels [47]. Alfalfa reportedly contains several useful phytochemicals, including phenolic compounds, saponins, and medicarpin, which possess inhibitory properties against several plant seedlings [48]. Other important phytocompounds extracted from this species include linoleic and linolenic acid, vitamins, and iron compounds [49,50]. Although the allelopathic properties of alfalfa on weeds have been documented, the exact nature of the allelopathic phytochemicals and their mechanisms of action remain poorly understood.

The main objective of this study was to explore the possible allelopathic effects of alfalfa extracts on callus growth in some weed species. Furthermore, TLC, HPLC, and NMR methods were used to determine the major allelochemicals present in the alfalfa leaf extracts. Additionally, the autotoxic compounds in alfalfa were determined. We also elucidated the mechanism involved in seed germination inhibition by alfalfa leaf extracts and assessed the correlation between the allelochemicals exuded by alfalfa and the rate of alfalfa seed inhibition. It is worth mentioning that, to our knowledge, this is the first report on the allelopathic effect of alfalfa on callus growth in different weeds.

## 2. Results

### 2.1. Effect of Alfalfa Leaf Extracts on Callus Induction

**Figure 1:**
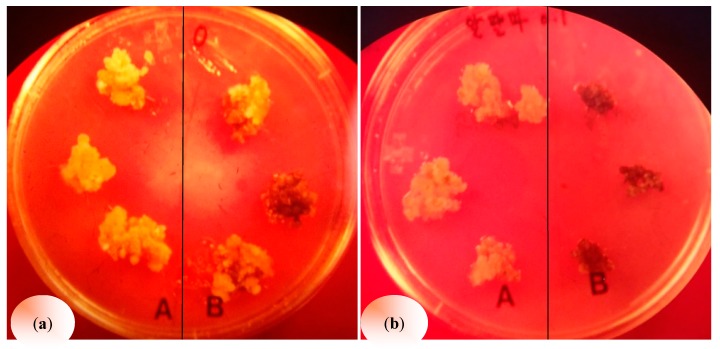
(**A**) Callus growth in the absence of alfalfa leaf extract. (**a**) Callus of *C. album*; (**b**) callus of *P. oleracea.* (**B**) Callus growth in the MS medium supplemented with 0.1% alfalfa leaf extract. (**a**) Callus of *C. album*; (**b**) callus of *P. oleracea*.

The application of alfalfa leaf extracts influenced callus induction and fresh weight in the studied weeds in a varied manner (Figure 1). Alfalfa leaf extracts (0.1 mg/mL) significantly promoted callus induction in *Digitaria ciliaris* (Table 1). However, increasing the concentration to 5 and 10 mg/mL, decreased the callus induction rate by twofold. All of the extract concentrations used decreased the callus induction rate in *Chenopodium album* in a concentration-dependent manner. The results also show that the callus of *C. album* was highly sensitive to 10 mg/mL alfalfa leaf extracts. The fresh weight of callus cultured in the Murashige and Skoog (MS) medium containing 5 mg/mL alfalfa leaf extract showed the highest suppression of callus induction in *Amaranthus lividus* and *Portulaca oleracea*. However, in these species, when the concentration of extracts was increased from 5 to 10 mg/mL, a reverse trend was observed. Similarly, the alfalfa leaf extracts inhibited the callus growth and reduced the callus fresh weight in *Commelina communis*.

### 2.2. Effect of Allelopathic Compounds on Callus Growth

To determine the relationship between allelopathy and the phytochemical constituents of alfalfa, its phenolic compound concentration and composition were determined using HPLC (Table 2). Six active allelopathic compounds were identified and quantified, with salicylic and *p*-hydroxybenzoic acids being the most predominant phenolic compounds, accounting for 80.8% of the all phenolic compounds (Table 2). In this study, calluses were treated with six standard phenolic compounds to measure their effect on callus growth in *P. oleracea*, *C. album*, and *Pinellia ternata*. All concentrations (10^−2^, 10^−3^, and 10^−5^ M) of all the tested phenolic compounds showed inhibitory effects on callus fresh and dry weights. In particular, the callus of *P. oleracea* was highly sensitive to these phenolic compounds, compared with the other species. The lower concentration of salicylic acid (10^−5^ M) was very active and effectively inhibited (26.90 ± 2.50 mg) the callus growth and fresh weight of *C. album*. Ferulic acid (10^−2^ M) had the highest effect on *P. oleracea*, represented by the lower fresh weight and fresh weight percentage (4.10 ± 0.30 mg and 0.60% ± 0.01%, respectively). Similarly, salicylic acid (10^−2^ M) highly inhibited the callus fresh weight and fresh weight percentage of *P. oleracea* and *C. album*. Increasing the ferulic acid concentration from 10^−5^ to 10^−3^ M had no effect on callus growth, as indicated by nonsignificant results (Table 3). Similar results were observed for *p*-coumaric and syringic acids.

### 2.3. Autotoxic Effect of Alfalfa Leaf Extracts

Alfalfa leaf extracts obtained using different fractions and concentrations exerted a significant effect on the alfalfa seed germination rate (Table 4). Most of the tested fractions exerted a concentration-dependent autotoxic effect on seed germination, total seedling weight, and seedling length. Aqueous alfalfa leaf extract fractions (10 mg/mL) showed the highest autotoxic effect, as indicated by a lower seed germination rate (50.40% ± 2.00%) with the lowest seedling total length (TL) and total weight (TW). The 80% MeOH extract and organic phase (acid hydrolysis) at a concentration of 10 mg/mL promoted seed germination, as represented by higher germination percentage (92.50% ± 4.00% and 92.50% ± 7.50%), compared to the control seeds.

### 2.4. Identification of Major Phenolic Compounds and Autotoxic Effects

The results show that quercetin (10^−5^ M) significantly inhibited alfalfa seed germination (57.30% ± 2.10%), seedling length (4.90 ± 0.10 cm), and seedling weight (1.42 ± 0.2 mg) (Table 5). Similarly, rutin exerted a low autotoxic effect on alfalfa seeds, as indicated by higher germination rates (82.30% ± 2.50%), seedling lengths (5.90 ± 0.20 cm), and seedling weights (1.69 ± 0.10 mg).

### 2.5. Identification of Major Saponins and Autotoxic Effects

**Figure 2:**
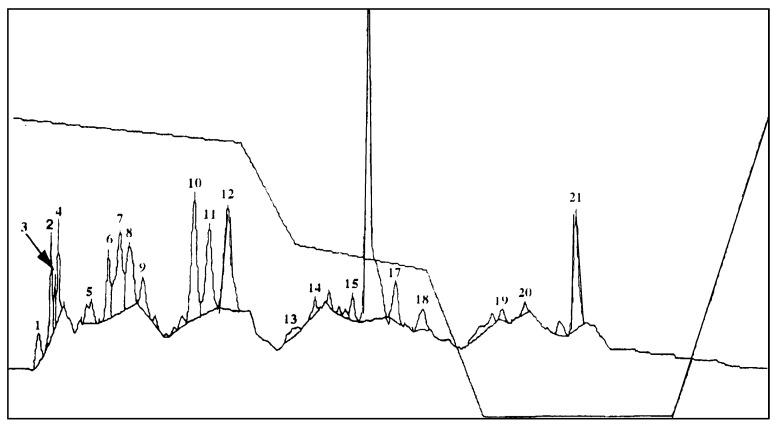
Chromatogram of alfalfa root parts–derived saponins. Peak 10: 3-Glc, 28 AraRhaXyl medicagenic acid (MA); peak 12: 3-Glc, 28-Glc MA; peak 16: 3-GlcA, 28 AraRhaXyl MA; peak 18: soyasaponin I; and peak 21: 3Glc MA.

**Figure 3:**
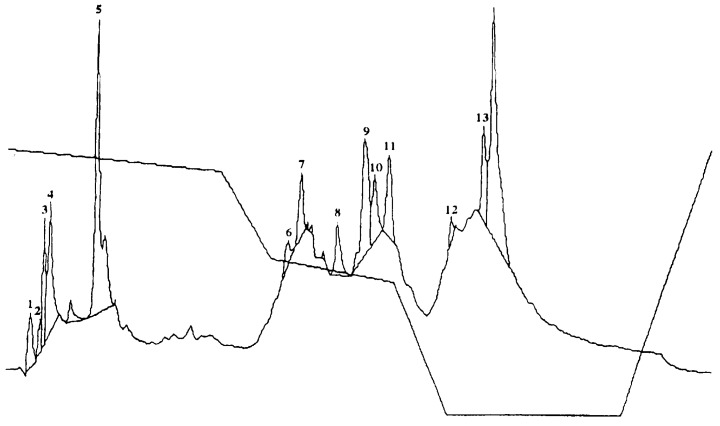
Chromatogram of alfalfa leaf-derived saponins. Peak 8: 3-GlcA, 28 AraRhaXyl medicagenic acid; peak 9: 3-GlcA, 28 AraRha medicagenic acid; and peak 11: Soyasaponin I.

**Figure 4:**
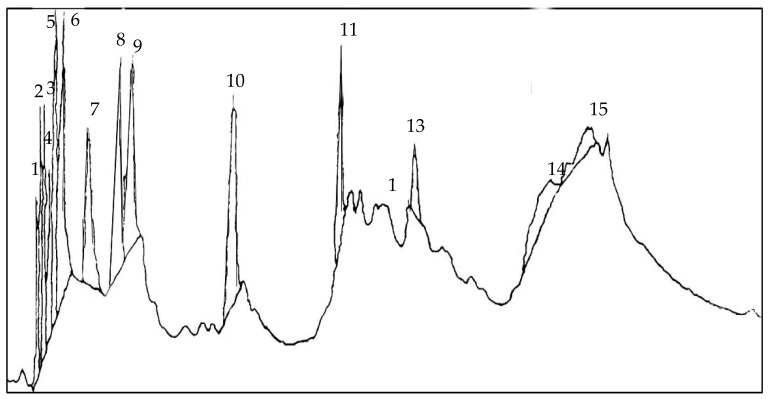
Chromatogram of prosaponin from alkaline hydrolysis of zanhic acid tridesmoside. Peak 11: zanhic acid.

**Figure 5:**
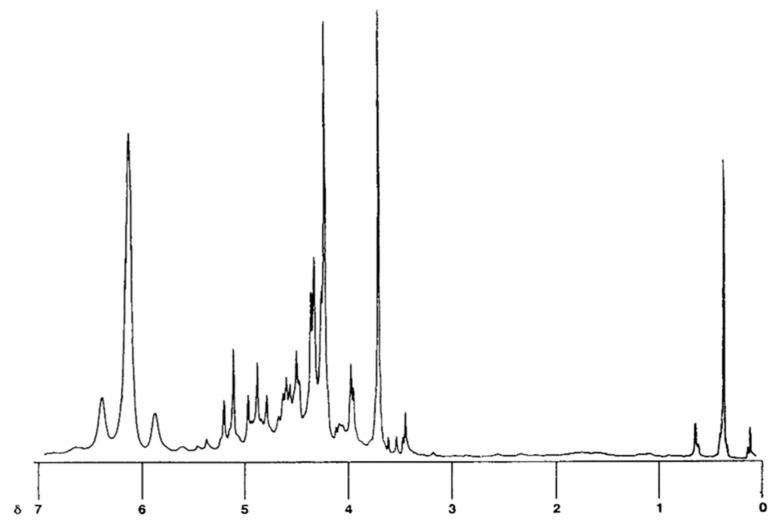
^1^H NMR spectrum of alfalfa-derived saponins.

**Figure 6:**
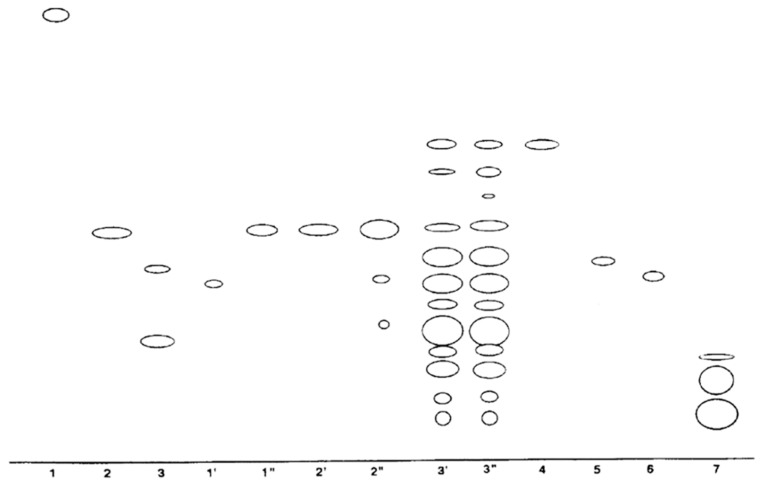
Thin-layer chromatography of crude extracts from alfalfa leaves, stems, and roots under UV light. 1: Medicagenic acid; 2: Soyasaponin 1; 3: 3-Glc-Glc, 28 AraRhaXyl medicagenic acid; 1′ and 1”: Stem samples; 2′ and 2”: Leaf samples; 3′ and 3”: Root samples; 4: 3-Glc, 28-Glc medicagenic acid; 5: 3-Glc, 28 AraRhaXyl medicagenic acid; 6: 3-GlcA, 28 AraRha medicagenic acid; and 7: Zanhic acid tridesmoside.

**Figure 7:**
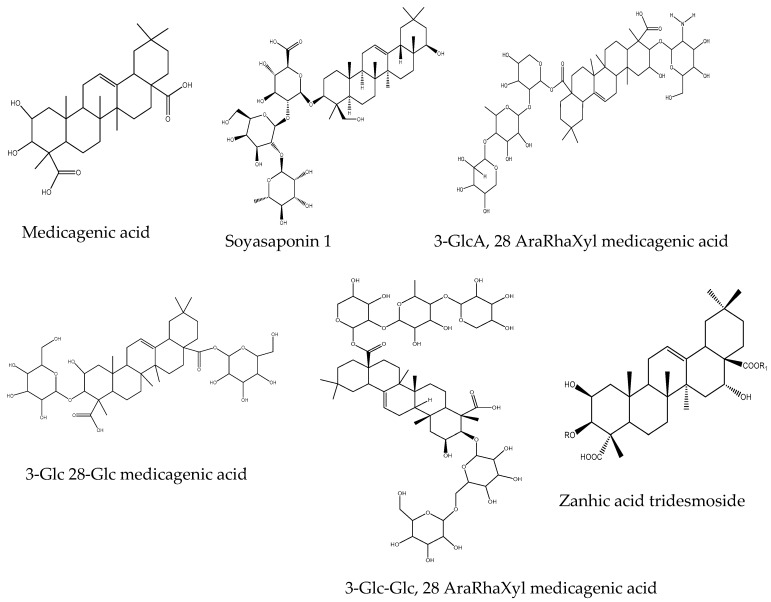
Structures of saponins identified in leaf extracts of alfalfa.

Autotoxic compounds of alfalfa were analyzed using HPLC methods (Figure 2, Figure 3 and Figure 4). Its seed germination rate showed a wide variation in the aliquot of different fractions. In this study, seven different saponins were identified from root and leaf extracts: Medicagenic acid; soyasaponin I; 3-Glc-Glc, 28 AraRhaXyl medicagenic acid; 3-Glc, 28-Glc medicagenic acid; 3-Glc, 28 AraRhaXyl medicagenic acid; 3-GlcA, 28 AraRha medicagenic acid; and zanhic acid tridesmoside (Figure 5, Figure 6 and Figure 7). These saponins were used to test autotoxicity by determining seed germination and seedling growth rates. The results showed that all saponin compounds influenced seed germination (Table 6). Among these, medicagenic acid showed the highest autotoxic effect, as represented by lower seed germination rates (68.7% ± 3.50%). No significant differences were found between the autotoxicity rates of 3-Glc, 28-Glc medicagenic acid and 3-GlcA, 28 AraRha medicagenic acid on alfalfa seed germination at a concentration of 500 ppm.

### 2.6. Principal Component Analysis (PCA)

**Figure 8:**
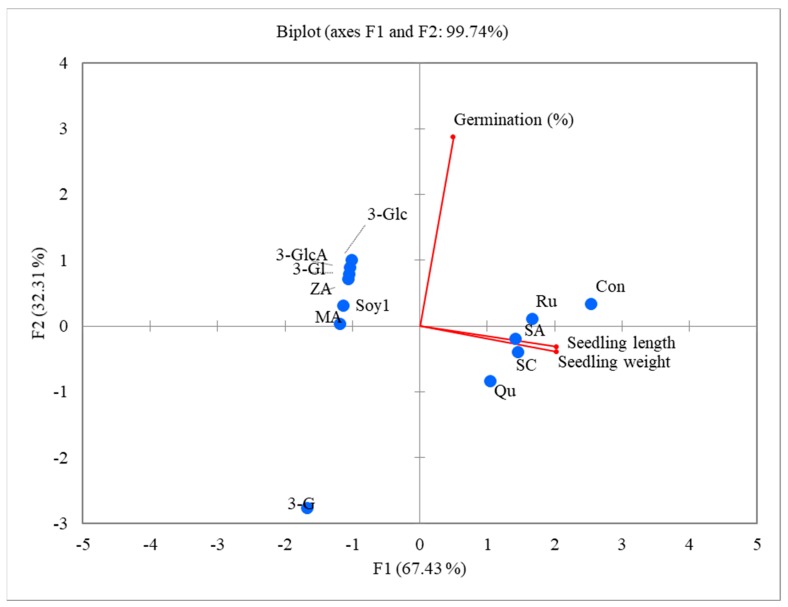
Principal composition analysis (PCA) biplot constructed by plotting the PC1 (vertical axis) scores against the PC2 (horizontal axis) scores obtained from phenolic compounds and saponins. MA: Medicagenic acid; Soy1: Soyasaponin I; 3G: 3-Glc-Glc, 28 AraRhaXyl medicagenic acid; 3-Gl: 3-Glc, 28-Glc medicagenic acid; 3-Glc: 3-Glc, 28 AraRhaXyl medicagenic acid; 3-GlcA: 3-GlcA, 28 AraRha medicagenic acid; ZA: Zanhic acid tridesmoside; Ru: Rutin; SA: Salicylic acid; SC: Scopoletin; Qu: Quercetin; and Con: Control.

To assess the correlation between the phenolic compounds and autotoxic properties of alfalfa, the GC data were subjected to PCA. PC1 (vertical axis) accounted for 67.58% of the total variance, whereas PC2 (horizontal axis) accounted for 32.16% (Figure 8). PCA showed higher loadings for 3-GlcA, 28 AraRhaXyl medicagenic acid, rutin, salicylic acid, and scopoletin. The direction and length of the arrow indicate the direction and magnitude of each variable’s contribution. The angle between the arrows and axis is inversely proportional to the correlation between each variable and the axis constructed in the ordination. The PCA results indicated that the phenolic compounds and saponin composition had significant effects on the variables. In this study, the variables oriented in the same direction were considered more closely related. The PCA analysis distinctly separated the alfalfa leaf extract–derived saponin compounds from the phenolic compounds. It is noteworthy to mention that all the variables (phenolic compounds) were located at the positive side (second and third quadrats) of PC1.

## 3. Discussion

In this study, the alfalfa leaf extracts showed a wide range of allelopathic effects on callus growth, seed germination, and seedling growth of various weeds. Callus growth and fresh weight were inversely related to extract concentration. Increasing the extract concentration from 0.1 to 5 mg/mL had an intense inhibitory effect on the biomass of the calluses of all studied weeds. The growth and development of the callus of *C. album* was highly sensitive to all the alfalfa extract concentrations, as indicated by smaller biomasses and higher callus growth inhibition rates. The lower alfalfa extract concentration (0.1 mg/mL) promoted callus growth in *D. ciliaris.* These results indicate that alfalfa leaf extract’s allelopathic effect on weeds is species specific. Moreover, some of the phenolic compounds present in alfalfa have been shown to exert both stimulatory and inhibitory effects on calluses [51]. Similar to the report by Braga et al. [52], we showed that higher concentrations of the identified allelochemicals effectively suppressed callus growth and decreased its biomass. In this study, the allelochemicals involved in the allelopathic effect of alfalfa were analyzed using HPLC. The identified phenolic compounds, including salicylic acid, syringic acid, ferulic acid, vanillic acid, *p*-coumaric acid, and *p*-hydroxybenzoic acid, significantly inhibited the callus growth of weeds in a concentration-dependent manner. The inhibitory effects of these compounds on callus biomass were observed with the 10^−5^ M alfalfa leaf extracts. Phenolic compounds such as *p*-coumaric acid reportedly inhibit photosynthesis and the enzymatic activities of PG1, CG6PDH, AID, and OPPP, which is detrimental to plant growth [53]. *p*-Coumarin and its derivatives reportedly induce water stress in plants by increasing osmolality and are known for their inhibitory effect on seed germination and plant growth [54]. Additionally, they may also exert a deleterious effect on root growth by altering the morphological and physiological structure of the root [55].

Moreover, some studies have suggested the direct involvement of reactive oxygen species (ROS) in allelopathy. For example, polyphenols are responsible for forming superoxide anions [56] and hydroxyl radicals by donating electrons to molecular oxygen [57], which, in turn, rapidly alters membrane permeability and cellular components such as DNA and proteins. In addition to their antioxidative properties, some phenolic compounds possess phytotoxic properties and cause excessive ROS regeneration, which affects plant growth and development; causes tissues injury, programmed cell death (PCD), and cell membrane disruption; and damages DNA and other biological molecules [58,59]. Some previous studies on the allelopathic properties of phenolic compounds have suggested that their phytotoxicity can have physiological effects such as the reduction of the activity of enzymes involved in ATP synthesis during photosynthesis [60,61,62]. Furthermore, these compounds may also possess the ability to alter mineral transportation and stomatal regulation in plants [63,64]. Moreover, an inhibitory effect of allelochemicals was attributed to their ability to deform the chloroplasts and mitochondria [62].

In order to assess autotoxicity, the inhibitory effect of the isolated saponins was studied in terms of alfalfa seed germination and visible seedling growth. The results indicated that alfalfa-derived saponins exert a wide range of inhibitory effects on alfalfa seed germination. It has been argued that the differences in the structures of the saponins may result in differences in their biological activities [65]. Moreover, the allelopathic potential of these compounds largely depends on the sugar groups attached to the aglycone and on the cultivars used [66,67]. The treated alfalfa seedlings experienced lack of root hairs, reduced seedling taproots, and complete root color loss. Amongst the isolated saponins, medicagenic acid most effectively suppressed alfalfa seedling growth, indicating that it is partially responsible for the autotoxic effect on alfalfa seedlings. Moreover, the autotoxic effects of zanhic acid glycosides and sapogenin salt have been reported by Oleszek and Jurzysta [66], who suggested that these effects are highly dependent on their concentration and structure [68]. Mung bean–derived soyasaponin I reportedly exerts a higher inhibitory effect on weed seedling elongation and root growth [69]. Moreover, although related, the other saponins (including medicagenic acid and monodesmosides) are more active than bidesmosides [59,70].

In this study, the presence of these compounds in the growing medium affected seed germination and seedling growth rates. Treatment of growing seedlings with saponins can cause structural changes in the cell membrane and cell wall swelling as well as alter the O_2_ permeability of the cell membrane, which ultimately decreases seedling growth [68]. Treatment with saponin has also been shown to alter water absorption at the lipid bilayer interface [71] and may affect cellulose synthesis [72]. High concentrations of quercetin and rutin have been shown to inhibit the seed germination of various plant species by impairing respiration and ATP levels in embryogenic cells via substrate oxidation or phosphate uptake inhibition, which may uncouple oxidative phosphorylation [73,74]. Additionally, treatment with rutin was shown to drastically reduce the protein content of *Arabidopsis* cells [75]. Zanhic acid, medicagenic acid, and soyasaponin reportedly caused browning of the meristematic area of seedling roots [70] and inhibited b-1,4-glucan synthesis, which is responsible for cellulose synthesis [72,76]. Although the synergistic activity of phenolic compounds and saponins was not investigated in the present study, it was reportedly observed in *Spodoptera littoralis* [77].

Studies showed that saponins present in the extracts of alfalfa are toxic to weeds and insects [78,79,80]. Medicagenic acid is the most effective allelochemical responsible for allelopathic potential of alfalfa. This compound is associated with reduction of insect activity and induced very high mortality in *Acyrthosiphon pisum* [81,82]. In another study, derivatives of medicagenic acids obtained from leaves extracts of alfalfa caused inhibition of plant growth of *Amaranthus, Lepidium,* and *Lycopersicon* sp. [83]. Moreover, other saponins such as zanhic acid tridesmoside, 3-GlcA, 28 AraRhaXyl medicagenic acid glycoside, and 3-GlcA, 28 AraRha medicagenic acid glycoside identified from alfalfa inhibited the reproduction and survival rate of aphids [78,84]. In a similar study, 3GlcA, 28 AraRhaXyl medicagenic acid obtained from the alfalfa extracts inhibited the larval growth and development of *Ostrinia nubilalis*, *Tenebrio molitor*, suppressed the growth of *Trichoderma viride* [79]. Data from the present study showed that alfalfa extracts with a high concentration of allelocompounds including phenolics, flavonoids, and saponins can provide valuable source of raw materials for the development of potential bioherbicides for weed control.

## 4. Materials and Methods

### 4.1. Chemicals

All chemicals and solvents used were of analytical grade and supplied by commercial providers. Methanol was obtained from J.T. Baker Chemical Co. (Phillipsburg, NJ, USA). Standards for phenolic compound and saponin estimation were purchased from Sigma-Aldrich Corp. (St. Louis, MO, USA).

### 4.2. Plant Materials

Seeds of the various weeds and alfalfa plants used in this study were kindly supplied by the Department of Applied Plant Science, Kangwon National University, South Korea.

### 4.3. Callus Regeneration and Subculture

Seeds of *C. album*, *A. lividus, D. ciliaris, P. oleracea*, and *C. communis* were sterilized with sodium hypochlorite (6%) for 30 min, rinsed by distilled water ≥6 times, dried using sterilized paper towels for 1 h, and then allowed to germinate in MS medium. Young leaves of in vitro grown plantlets of *C. album*, *A. lividus, D. ciliaris, P. oleracea,* and *C. communis* were used for callus induction, using full strength Murashige and Skoog (MS) medium. MS medium was prepared by mixing 3% (*w/v*) sucrose supplemented with 1 mg L^−1^ 2,4-dichloroohenoxyacetic acid (2,4-D). The pH of the medium was adjusted to 5.8 using I N NaOH and HCl. This medium was gelled using 0.8% plant agar before autoclaving at 1.1 kg cm^−2^ for 15 min. Sterilized medium (20 mL) was dispensed into each Petri dish (10 × 15 cm) and used for inducing calluses. Young leaves from the in vitro grown plants were cut across the midrib and immediately placed in the callus-induction medium. Regenerated calluses were proliferated further by subculturing every week in the MS medium supplemented with 1 mg L^−1^ 2,4-D.

### 4.4. Evaluation of Allelopathic Effects of Plant Extracts on Callus Growth

Calluses induced in the leaf section of different weeds were cultured in MS medium supplemented with 1 mg L^−1^ 2,4-D. Fresh and healthy callus (50 mg) from each plant sample were soaked in different alfalfa leaf extract concentrations (0.1%, 5%, and 10% *w/v*). Six to eight calluses were grown in each Petri dish containing MS medium supplemented with 1 mg L^−1^ 2,4-D and plant extracts. The Petri dishes were allowed to grow in a culture room for four weeks and three replications were maintained for each treatment. For the control treatment, the plant extracts were replaced with distilled water.

### 4.5. Effect of Allelopathic Compounds on Callus Growth

To assess the effect of allelopathic compounds on growth, the identified phenolic compounds, including salicylic acid, *p*-coumaric acid, vanillic acid, *p*-hydroxybenzoic acid, ferulic acid, and syringic acid at concentrations of 10^−2^, 10^−3^, and 10^−5^ M, were prepared. They were then mixed with MS medium supplemented with 1 mg L^−1^ 2,4-D, upon which six to eight young and fresh calluses were placed and maintained at 25 ± 1 °C for 40 d. The callus fresh weight was recorded after 40 d.

### 4.6. Autotoxic Effect of Alfalfa Leaf Extracts

Alfalfa seeds were sterilized using sodium hypochlorite (6%) for 30 min, rinsed with distilled water ≥6 times, and then dried using sterilized paper towels for 1 h. A sterilized filter paper containing 100 seeds was placed in a Petri dish and 20 mL of different concentrations of phenolic compound solution was added, while the same amount of distilled water was used for the control. Treated seeds were transferred to the growth chamber at 25 ± 1 °C, and the germination percentage of each species was determined weekly. The fresh weight of the germinated seedlings was also recorded. The seedlings were then oven dried at 60 °C for 5 h, and their dry weight recorded.

Percentage inhibition (%) = 1 − (plant extracts/control) × 100.(1)

### 4.7. Preparation of Plant Extracts

To assess the composition and concentration of its allelochemical compounds, alfalfa leaves were collected from the experimental field of Kangwon National University (South Korea) in 2014, 2015, and 2016. The collected leaves samples were dried at room temperature (25 °C) for 24 h. Then, 10 g of the ground, dried powder samples of each plant were each dissolved in 80% ethanol, using a 100-mL conical flask with continuous shaking (40 rpm) at room temperature for 20 h. The solutions were then filtered using Whatman No. 1 filter paper for debris removal. The obtained filtrates were evaporated to dryness in a rotary evaporator (Eyela, SB-1300, Shanghai Eyela Co. Ltd.) at 42 °C. The dry filtrate residues were then re-dissolved in 80% HPLC-grade ethanol (0.01 mL) for phenolic compound analysis using HPLC.

### 4.8. Phenolic Compound Identification and Quantification Using HPLC

The identification and quantification of phenolic compounds from different accessions were carried out using HPLC, as previously described [85]. Briefly, 1 g of each dried seed sample was crushed to powder, extracted with 10 mL of 80% methanol, and shaken in an orbital shaker for 24 h at room temperature. To remove debris, all sample solutions were filtered using a Whatman No. 1 filter paper. The extracted solvent (80% methanol) was then evaporated at 40 °C using a vacuum rotary evaporator. The dried extract was dissolved in 80% methanol (10 mL) to obtain a 50 µg/mL solution, passed through a 0.45 µm filter unit (TITAN syringe filter nylon membrane), and then injected into the HPLC system. Quantification of individual phenolic compounds in each sample was performed using the HPLC system from Shimadzu Corporation (Kyoto, Japan), equipped with two LC-10AD VP pumps and a SPD-M10A VP photodiode array detector (280 nm). The YMC-Pack ODS-AM-303 column (4.6 mm id × 250 mm, 5 μm particle size) was used for chromatographic separation of phenolic compounds. The injection volume, flow rate, and detection wavelength were 20 µL, 1 mL min^−1^, and 280 nm, respectively. The mobile phase consisted of solvent A (0.1% glacial acetic acid adjusted with water) and solvent B (0.1% glacial acetic acid in acetonitrile). Gradient elution was as follows: Solvent B was elevated from 8% to 10% B (2 min), 10–30% B (27 min), 30–90% B (50 min), 90–100% B (52 min), and then held at 100% of B (57 min). Phenolic compound calibration curves were obtained using different concentrations (10, 50, and 100 ppm) of standard compounds. Polyphenols were identified and quantified by matching their retention times with those of authentic phenolic compound standards.

### 4.9. Identification of Major Saponins in Alfalfa Leaves

Fresh young alfalfa leaves were harvested, dried, and ground at room temperature. Approximately 2 g of the powdered sample was boiled in 50 mL of methanol-distilled water (4:1) for 6 h. To remove debris, all the sample solutions were filtered through Whatman No. 1 filter paper. The extracted solvent (80% methanol) was evaporated at 40 °C in a vacuum rotary evaporator. The extract was dissolved in ether to precipitate the saponins, at room temperature. The obtained solution was centrifuged at 4 °C for 20 min. Purification of saponins in the precipitate sample was performed as previously described [86]. Briefly, 10 g of crude saponin samples were suspended in 100 mL CHCl. Thin layer chromatography (TLC) was performed to visualize the patterns of unknown compounds. After repeated chromatography, compound identification in the samples was performed using NMR analysis.

### 4.10. Nuclear Magnetic Resonance (NMR) Spectroscopic Analyses

For structural analysis, ^1^H and ^13^C spectra were measure on an NMR spectrometer Varian Unity (Varian Inc., Palo Alto, CA, USA), 300 MHz, d6-DMSO, with tetramethylsilane (TMS) as an internal standard. Deuterated chloroform (CDCl_3_) was used as the solvent.

### 4.11. Statistical Analyses

Each experiment was performed in triplicate, and the data are expressed as mean ± standard deviation. Quantitative data were statistically analyzed using one-way analysis of variance (ANOVA). Significant differences between the obtained data were determined using Duncan’s multiple range tests, with *p* < 0.05 implying statistical significance. All statistical analyses were performed using SPSS v20.0 from SPSS Inc. (Chicago, IL, USA). Correlated variables were identified using Pearson’s correlation coefficient. The principal component analysis (PCA) of quantitative morphological traits was also performed using SPSS software v20.0.

## 5. Conclusions

In conclusion, callus growth in the weed species was highly sensitive to the alfalfa leaf extracts-derived allelopathic phytochemicals. Additionally, all identified saponins possessed autotoxic properties, with the autotoxicity of medicagenic acid being the most potent. Moreover, the significant positive correlation between seed germination rate and phenolic compound concentration indicates that these compounds have a higher inhibitory effect on callus induction and postemergence of weeds and can be potentially developed as bioherbicides for organic farming, which will help conserve biodiversity and the environment.

## Figures and Tables

**Table 1:** Effect of alfalfa leaf extracts on callus fresh weight.

Treatment	Concentration (%).	Callus Fresh Weight (mg)				
		*Digitaria ciliaris*	*Chenopodium album*	*Amaranthus lividus*	*Portulaca oleracea*	*Commelina communis*
Control		230.50 ± 3.50 ^b^	1130.18 ± 10.00 ^d^	250.10 ± 8.00 ^c^	1120.11 ± 15.00 ^d^	1130.20 ± 12.00 ^d^
MS + Alfalfa	0.1	345.50 ± 4.00 ^c^	819.30 ± 10.50 ^c^	125.50 ± 4.00 ^b^	610.40 ± 5.00 ^c^	118.70 ± 5.90 ^c^
	5	115.44 ± 5.00 ^a^	6.20 ± 0.40 ^b^	100.50 ± 2.50 ^a^	100.80 ± 6.80 ^a^	99.40 ± 3.90 ^b^
	10	115.40 ± 4.00 ^a^	5.20 ± 0.50 ^a^	125.50 ± 5.00 ^b^	105.80 ± 5.90 ^b^	59.90 ± 2.00 ^a^

Data are expressed as mean ± standard deviation (*n* = 3). Data with the same letter in a column are not significantly different, as determined using Duncan’s multiple range test (*p* < 0.05). MS, Murashige and Skoog medium.

**Table 2:** Phenolic compounds in the alfalfa leaf extracts.

Name of Phenolic Compounds	Phenolic Compound Concentration (µg/mL)
Salicylic acid	1927.01 ± 12.50 ^f^
*p*-Hydroxybenzoic acid	372.01 ± 5.00 ^e^
Vanillic acid	137.50 ± 6.50 ^c^
Syringic acid	50.00 ± 3.40 ^a^
*p*-Coumaric acid	171.50 ± 5.80 ^d^
Ferulic acid	105.00 ± 3.80 ^b^
Total	2762.50 ± 13.50 ^g^

Data are expressed as mean ± standard deviation (*n* = 3). Different small letters indicate a significant difference as determined using Duncan’s multiple range test (*p* < 0.05).

**Table 3:** Effect of standard phenolic compounds on callus fresh weight.

Chemical	Concentration (M)	*Portulaca oleracea*		*Chenopodium album*		*Pinellia ternata*	
		Fresh Weight (mg)	Fresh Weight (%)	Fresh Weight (mg)	Fresh Weight (%)	Fresh Weight (mg)	Fresh Weight (%)
Salicylic acid	10^−5^	140.10 ± 4.00 ^n^	19.80 ± 0.90 ^m^	26.90 ± 2.50 ^i^	18.50 ± 1.50 ^i^	160.20 ± 5.50 ^n^	64.10 ± 1.50 ^n^
	10^−3^	30.50 ± 2.00 ^g^	4.20 ± 0.50 ^g^	11.70 ± 1.00 ^e^	8.10 ± 0.50 ^f^	82.30 ± 2.00 ^i^	32.90 ± 1.00 ^i^
	10^−2^	7.20 ± 0.50 ^d^	1.00 ± 0.10 ^d^	5.70 ± 0.20 ^b^	3.90 ± 0.20 ^c^	40.20 ± 1.50 ^f^	16.10 ± 1.00 ^f^
Syringic acid	10^−5^	193.30 ± 5.90 ^p^	27.30 ± 2.00 ^n^	45.90 ± 2.00 ^l^	3.20 ± 0.40 ^b^	187.00 ± 11.00 ^p^	74.80 ± 4.00 ^p^
	10^−3^	48.20 ± 2.50 ^i^	6.80 ± 0.50 ^i^	45.20 ± 4.00 ^k^	3.20 ± 0.20 ^b^	42.30 ± 2.00 ^g^	16.90 ± 1.50 ^g^
	10^−2^	4.40 ± 0.50 ^b^	0.60 ± 0.01 ^a^	3.70 ± 0.20 ^a^	2.60 ± 0.10 ^a^	3.80 ± 0.50 ^a^	1.80 ± 0.01 ^a^
Ferulic acid	10^−5^	286.30 ± 10.50	4.00 ± 0.50 ^o^	82.40 ± 4.00 ^n^	56.70 ± 3.00 ^l^	190.00 ± 10.00 ^q^	76.00 ± 4.00 ^q^
	10^−3^	45.80 ± 2.00 ^h^	6.50 ± 1.00 ^h^	94.70 ± 5.00 ^p^	65.20 ± 2.00 ^p^	84.20 ± 3.00 ^k^	33.70 ± 2.00 ^k^
	10^−2^	4.10 ± 0.30 ^a^	0.60 ± 0.01 ^a^	8.60 ± 0.50 ^c^	5.90 ± 0.30 ^d^	15.30 ± 1.50 ^c^	6.10 ± 0.10 ^c^
Vanillic acid	10^−5^	93.40 ± 3.50 ^l^	13.20 ± 1.00 ^k^	128.50 ± 2.00 ^o^	88.50 ± 4.00 ^o^	166.00 ± 3.00 ^o^	66.40 ±2.00 ^o^
	10^−3^	90.70 ± 4.50 ^k^	12.80 ± 1.00 ^j^	41.80 ± 1.00 ^j^	28.80 ± 2.00 ^j^	82.00 ± 2.00 ^j^	32.60 ± 0.20 ^j^
	10^−2^	6.20 ± 1.00 ^c^	0.90 ± 0.10 ^c^	10.60 ± 0.50 ^d^	7.30 ± 0.50 ^e^	5.40 ± 0.40 ^b^	2.20 ± 0.10 ^b^
*p*-Coumaric acid	10^−5^	95.60 ± 5.00 ^m^	13.50 ± 1.00 ^l^	82.80 ± 5.00 ^n^	57.00 ± 3.00 ^m^	85.50 ± 3.00 ^l^	34.20 ± 2.00 ^l^
	10^−3^	23.20 ± 2.00 ^f^	3.30 ± 0.50 ^f^	14.30 ± 1.00 ^g^	9.90 ± 0.80 ^g^	32.10 ± 2.00 ^e^	12.80 ± 1.00 ^e^
	10^−2^	21.80 ± 2.00 ^e^	3.10 ± 0.50 ^e^	13.10 ± 0.80 ^f^	9.00 ± 1.00 ^g^	20.50 ± 2.00 ^d^	8.20 ± 0.50 ^d^
*p*-Hydroxybenzoic acid	10^−5^	183.50 ± 6.80 ^o^	26.0 ± 2.50 ^n^	87.50 ± 2.00 ^o^	60.30 ± 3.00 ^n^	102.30 ± 6.50 ^m^	40.90 ± 3.00 ^m^
	10^−3^	82.20 ± 3.90 ^j^	11.60 ± 1.00 ^j^	78.70 ± 3.00 ^m^	54.20 ± 4.00 ^k^	53.50 ± 2.00 ^h^	21.40 ± 1.50 ^h^
	10^−2^	22.60 ± 2.00 ^f^	3.20 ± 0.50 ^e^	20.00 ± 1.00 ^h^	13.80 ± 1.00 ^h^	21.30 ± 1.00 ^d^	8.50 ± 0.50 ^d^
Control	-	707.8 ± 15.60 ^q^	-	145.2 ± 5.00 ^q^		250.0 ± 8.00 ^r^	

Experimental data are expressed as mean ± standard deviation (*n* = 3). Different small letters indicate a significant difference at *P* < 0.05. Data with the same letter in a column are not significantly different, as determined using Duncan’s multiple range test.

**Table 4:** Effect of various alfalfa leaf extract fractions on alfalfa seed germination.

Fraction	Concentration (mg/mL)	Germination Percentage (%)	* TL (cm)	** TW (mg)
Control		90.00 ± 3.50 ^p^	8.60 ± 1.00 ^q^	2.04 ± 0.01 ^j^
80% MeOH extract	5	92.50 ± 4.00 ^r^	8.40 ± 1.10 ^o^	2.50 ± 0.02 ^j^
	10	68.01 ± 2.00 ^k^	5.70 ± 0.50 ^l^	1.62 ± 0.01 ^g^
Organic phase (nonpolar compounds)	5	85.50 ± 5.00 ^o^	6.50 ± 0.40 ^m^	1.79 ± 0.01 ^h^
	10	65.30 ± 4.00 ^i^	5.10 ± 0.30 ^h^	1.54 ± 0.01 ^f^
Aqueous phase	5	64.50 ± 3.90 ^g^	4.90 ± 0.20 ^g^	1.29 ± 0.01 ^c^
	10	50.40 ± 2.00 ^a^	3.50 ± 0.11 ^a^	1.01 ± 0.01 ^a^
Organic phase (aglycones)	5	90.10 ± 3.50 ^q^	8.30 ± 1.00 ^n^	1.96 ± 0.02 ^i^
	10	60.60 ± 4.59 ^f^	4.10 ± 0.40 ^d^	1.33 ± 0.01 ^d^
Aqueous phase (glycosides)	5	79.50 ± 5.00 ^n^	6.50 ± 0.60 ^m^	1.58 ± 0.01 ^f^
	10	76.50 ± 3.00 ^m^	5.60 ± 0.20 ^k^	1.50 ± 0.01 ^f^
Organic phase (alkaline hydrolysis)	5	79.50 ± 6.00 ^n^	5.30 ± 0.40 ^i^	1.75 ± 0.01 ^h^
	10	53.60 ± 3.00 ^c^	3.60 ± 0.11 ^b^	1.16 ± 0.01 ^b^
Aqueous phase (alkaline hydrolysis)	5	65.00 ± 3.50 ^h^	4.90 ± 0.20 ^g^	1.46 ± 0.01 ^e^
	10	54.50 ± 2.50 ^d^	3.70 ± 0.30 ^c^	1.25 ± 0.01 ^c^
Organic phase (acid hydrolysis)	5	92.50 ± 7.50 ^r^	8.50 ± 0.90 ^p^	2.04 ± 0.02 ^j^
	10	65.70 ± 5.00 ^j^	4.80 ± 0.10 ^f^	1.50 ± 0.01 ^f^
Aqueous phase (acid hydrolysis)	5	68.50 ± 3.50 ^l^	5.50 ± 0.40 ^j^	1.54 ± 0.01 ^f^
	10	53.50 ± 0.05 ^b^	4.70 ± 0.40 ^e^	1.33 ± 0.01 ^d^

Experimental data are expressed as mean ± standard deviation (*n* = 3). Different small letters indicate a significant difference at *P* < 0.05. Data with the same letter in a column are not significantly different, as determined using Duncan’s multiple range test. * TL, total length; ** TW, total weight.

**Table 5:** Autotoxic effect of HPLC-identified compounds on alfalfa seed germination and seedling growth.

Phenolic Compound	Germination Percentage (%)	Seedling Length (cm)	Seedling Weight (mg)
Salicylic acid	74.00 ± 2.00 ^c^	5.40 ± 0.20 ^b^	1.60 ± 0.1 ^b^
Scopoletin	69.50 ± 1.50 ^b^	5.60 ± 0.30 ^c^	1.63 ± 0.1 ^c^
Rutin	82.30 ± 2.50 ^d^	5.90 ± 0.20 ^d^	1.69 ± 0.1 ^d^
Quercetin	57.30 ± 2.10 ^a^	4.90 ± 0.10 ^a^	1.42 ± 0.2 ^a^
Control	91.30 ± 1.50 ^e^	8.40 ± 0.50 ^e^	1.98 ± 0.1 ^e^

Experimental data are expressed as mean ± standard deviation (*n* = 3). Different small letters indicate a significant difference at *P* < 0.05. Data with the same letter in a column are not significantly different, as determined using Duncan’s multiple range test.

**Table 6:** Identification of saponins in the alfalfa leaf extracts.

Saponin	Concentration (ppm)	Germination (%)
Medicagenic acid	500	68.7 ± 3.50 ^b^
Soyasaponin I	500	75.6 ± 6.80 ^c^
3-Glc-Glc, 28 AraRhaXyl medicagenic acid	500	0 ^a^
3-Glc, 28-Glc medicagenic acid	500	87.3 ± 5.50 ^e^
3-Glc, 28 AraRhaXyl medicagenic acid	500	92.4 ± 8.90 ^f^
3-GlcA, 28 AraRha medicagenic acid	500	86.7 ± 6.50 ^e^
Zanhic acid tridesmoside	500	85.4 ± 5.00 ^d^

Data are expressed as mean ± standard deviation (*n* = 3). Different small letters indicate a significant difference at *P* < 0.05. Data with the same letter in a column are not significantly different, as determined using Duncan’s multiple range test.
